# Accidental Cutaneous Burns Secondary to Salbutamol Metered Dose Inhaler

**DOI:** 10.1155/2010/201809

**Published:** 2010-12-27

**Authors:** Ashutosh Kale, Fiona Shackley

**Affiliations:** Department of Pediatric Medicine, Sheffield Children's Hospital, Sheffield S10 2TH, UK

## Abstract

We report a case of accidental cutaneous burns caused by salbutamol metered dose inhaler. A 9-year-old boy underwent dental extraction at a children's hospital and was incidentally noted to have burn injuries on dorsum of both hands. On questioning, the boy revealed that a few days ago his 14-year-old brother, who is an asthmatic, playfully sprayed his salbutamol metered dose inhaler on the back of both his hands with the inhaler's mouth piece being in direct contact with the patient's skin. On examination, there was a rectangular area of erythema with superficial peeling on the dorsum of both hands, the dimensions of which exactly matched those of the inhaler's mouthpiece. It is possible that the injury could have been a chemical burn from the pharmaceutical/preservative/propellant aerosol or due to the physical effect of severe cooling of the skin or mechanical abrasive effect of the aerosol blasts or a combination of some or all the above mechanisms. This case highlights the importance of informing children and parents of the potentially hazardous consequences of misusing a metered dose inhaler.

A 9-year-old boy underwent dental extraction at a children's hospital and was incidentally noted to have burn injuries on dorsum of both hands. Pediatric team was contacted to exclude nonaccidental injury.

On questioning, the boy revealed that a few days ago his 14-year-old brother, who is an asthmatic, playfully sprayed his salbutamol metered dose inhaler on the back of both his hands with the inhaler's mouth piece being in direct contact with the patient's skin. The boy did not feel anything unusual immediately afterwards but noted redness in the sprayed areas the next morning. As the patient and his brother feared punishment from their mother, they did not tell anyone about the above.

On examination, there was a rectangular area of erythema with superficial peeling on the dorsum of both hands, the dimensions of which exactly matched those of the inhaler's mouthpiece (Figures 1 and 2). The boy did not have any other injuries, and he was interacting appropriately with his mother. Neither the child nor his family was known to the social services, and there were no previous child protection issues.

Once we were satisfied that this was an accidental injury, the pharmaceutical company which manufactured the inhaler was contacted. The pharmaceutical company was not aware of salbutamol causing cutaneous burn injuries. 

On reviewing the literature, a case of a 22-year-old asthmatic with history of mental illness presenting with self-inflicted cutaneous burn injuries due to salbutamol inhaler has been reported [1]. A 14-year-old girl with self-induced areas of hypo- and hyperpigmentation on her forearm as a result of applying ten blasts of an asthmatic aerosol inhaler directly to her skin has also been reported [2]. Similar salbutamol inhaler-induced burn injuries in children have been reported by Patel and Potter [3] and Arun et al. [4].

It is possible that the injury could have been a chemical burn from the pharmaceutical/preservative/propellant aerosol, due to the physical effect of severe cooling of the skin, mechanical abrasive effect of the aerosol blasts, or a combination of some or all the above mechanisms [1].

It is important that children and parents be informed of the potentially hazardous consequences of misusing a metered dose inhaler.

## Figures and Tables

**Figure 1:**
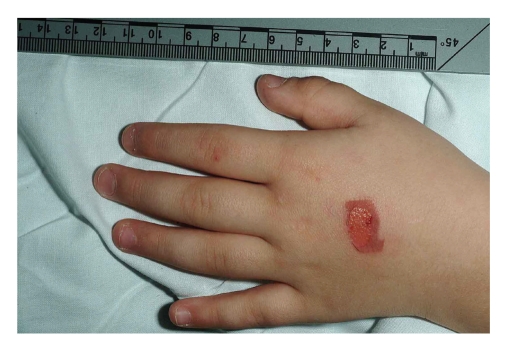
Salbutamol inhaler-induced burn (left hand).

**Figure 2:**
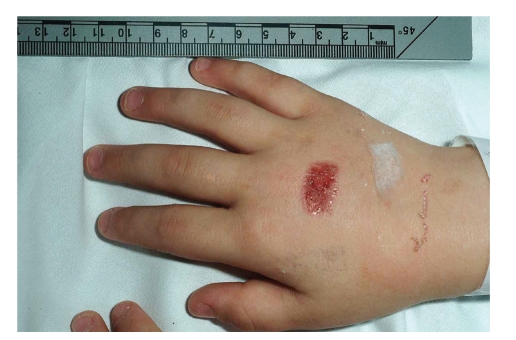
Salbutamol inhaler-induced burn (right hand).
